# Supplementary material for: Rapid antithrombin assay from human blood plasma utilizing smartphone-based flow observation on paper chips
Source: PLOS Digit Health. 2026 Feb 10;5(2):e0001209. doi: 10.1371/journal.pdig.0001209 (PMC12890117; doi:10.1371/journal.pdig.0001209)
Supplement: Attachment — Submitted filename: PDIG-D-25-00557-Reiewer recommendation.docx [file pdig.0001209.s009.docx]

**Reviewer Report**

**Title: Point of Care Antithrombin III Assay from Human Blood Plasma Utilizing Smartphone-Based Flow Observation on Paper Chips**

**Manuscript Number: PDIG-D-25-00557**

**Journal: PLOS Digital Health**

**General Assessment**

This manuscript presents a technically innovative proof-of-concept for detecting Antithrombin III (ATIII) using smartphone-based flow velocity measurements on paper microfluidic chips. While the concept is novel and potentially impactful, the current study does not sufficiently support its translation into a point-of-care (POC) diagnostic tool.

**Major Comments**

**1. POC Applicability and Validation**

The authors claim that this assay could fill a gap in POC testing for ATIII. However, this claim is not well substantiated:

- The assay is **not validated against the immunoassays currently used in clinical diagnostics**, such as Immunoassays/ELISA or functional activity assays.
- There is **no formal method comparison** (e.g., Bland–Altman analysis, regression, or bias estimation) between the smartphone-based assay and the ELISA results.
- The **clinical relevance and interchangeability** of the smartphone-based flow assay with existing diagnostic standards remain unclear.

**Recommendation**: Include a direct method comparison using patient samples across the clinical range, with appropriate statistical analysis of agreement and bias.

**2. Claim of Filling a Diagnostic Gap**

The manuscript suggests that this method fills a critical gap in ATIII testing. However, this is not convincing:

- Portable immunoassay platforms (e.g., lateral flow, microfluidic ELISA) are already being developed for multiple analytes and are more directly translatable to POC settings.
- The proposed method requires precise dilution, controlled environmental conditions, and smartphone-based video analysis, which may not be feasible in real-world point-of-care (POC) environments typically operated by non-laboratorians.

**Recommendation**: Temper the claim and discuss alternative POC strategies. Justify why this method offers a unique advantage over portable immunoassay devices.

**3. LoD Determination and Method Evaluation**

The manuscript mentions sensitivity and detection limits but does not describe how **Limit of Detection (LoD)** was formally determined.

- There is **no mention of CLSI guidelines** (e.g., EP17-A2, EP05-A3, EP09-A3) or any standard framework for evaluating analytical performance.
- LoD appears to be inferred from calibration curves, but without statistical justification or replicate data.

**Recommendation**:

- Clearly define how LoD was calculated (e.g., blank + 1.645 × SD of low concentration sample).
- State whether CLSI or other recognized guidelines were consulted.
- Discuss precision, reproducibility, and analytical range in the context of clinical diagnostics.

Additionally, the manuscript should address how this method compares to **in vitro diagnostic standards** in terms of:

- Analytical sensitivity and specificity
- Bias and agreement
- Sample preparation complexity
- Turnaround time and operational feasibility

**4. ELISA Kit Details (Line 219)**

The manuscript references the use of a commercial ELISA kit to determine endogenous ATIII levels but lacks sufficient detail.

**Recommendation**: Provide:

- Full kit name, manufacturer, and catalog number (already partially listed as SimpleStep Human ATIII ELISA, Abcam).
- Lot number, calibration range, and sensitivity.
- Sample preparation and dilution protocols.
- Whether ELISA was used as a reference method for validation.

**5. Device Translation and Usability**

The manuscript does not address how the smartphone-based flow assay would be **translated into a deployable device** for clinical use.

**Recommendation**:

- Describe the envisioned device architecture (e.g., integrated chip-reader, automated sample loading).
- Discuss challenges in standardizing flow velocity measurements across different smartphones and environmental conditions.
- Address reproducibility and robustness in field settings.

**Minor Comments**

- **Figures**: Consider adding a schematic comparing the smartphone-based assay to a conventional immunoassay workflow.
- **Terminology**: Clarify units and dilution factors consistently (e.g., ng/mL vs mg/dL).
- **Data Presentation**: Include calibration curves and raw flow velocity data in supplementary materials.

**Conclusion**

This manuscript presents an interesting and technically sound proof-of-concept. However, its current form does not support the claim of clinical POC applicability. Additional validation, comparative analysis, and discussion of device translation are needed to strengthen the manuscript and justify its relevance to clinical diagnostics.

**Final Note**: The title should be revised to remove the term “Point of Care,” as the current assay is not yet suitable for POC use. POC testing is typically performed by non-laboratorians, and ease of use, minimal sample preparation, and straightforward data interpretation are essential. These aspects are not adequately addressed in the current study.
