# Supplementary material for: Rapid antithrombin assay from human blood plasma utilizing smartphone-based flow observation on paper chips
Source: PLOS Digit Health. 2026 Feb 10;5(2):e0001209. doi: 10.1371/journal.pdig.0001209 (PMC12890117; doi:10.1371/journal.pdig.0001209)
Supplement: Attachment — Submitted filename: Response.docx [file pdig.0001209.s011.docx]

**RESPONSE LETTER (PDIG-D-25-00557)**

**Rapid Antithrombin Assay from Human Blood Plasma Utilizing Smartphone-Based Flow Observation on Paper Chips**

**Ashley K. Mathews, Allison J. Eby, Jocelyn Reynolds, Raymond K. Wong, and Jeong-Yeol Yoon**

**Additional Editor Comments:**

The term "antithrombin III (ATIII)" has largely been replaced by antithrombin (AT) in the clinical literature (e.g., J Thromb Haemost. 2020 Jan;18(1):17-22. doi: 10.1111/jth.14648.) I would advise that the authors update their nomenclature to reflect this.

Response: We changed them to antithrombin and AT throughout the manuscript, including all figures.

The sentence "Individuals who are predisposed to thrombotic events are likely to be ATIII-deficient, which could be hereditary or acquired over time," is confusing as there are indeed many other reasons one might be predisposed to thrombotic events outside of AT deficiency. - Reviewer #1 also points this out.

Response: The sentence was removed to avoid confusion. The new sentence directly discusses AT deficiency from the beginning:

… ~~Individuals who are predisposed to thrombotic events are likely to be ATIII-deficient, which could be hereditary or acquired over time.~~ AT deficiency could be hereditary or acquired over time. …

Limitations section should make clear that this assay aims to replace quantitative AT assays but would not replace AT activity assays which are required to diagnose qualitative defects in AT function.

Response: Added this limitation to the Discussion:

Fourthly, this assay aims to replace quantitative AT assays but would not replace AT activity assays. Such AT activity assays are required to diagnose qualitative defects in AT function, which is not the aim of this study.

The title should be revised as per Reviewer #2's comment given significant barriers to POC application at present; Reviewer #1 also points to current limitations in translating current assay to POC test.

Response: We removed most of the “Point of Care” from the title, abstract, keywords, and main text to avoid overstatement.

Also, please refer to our responses to reviewer #2’s comment 2 and reviewer #1’s comment 3.

**Reviewer #1:**

The authors detail a method for determining AT levels at the bedside over using traditional laboratory instrumentation. This manuscript describes proof of principle for determination of AT levels in a POC device.

1. The authors use PBS and human blood for these experiments and note differences between the two as expected as the authors discuss in the text. The addition of additional medications in patients that are undergoing procedures in hospitals present challenges for laboratory instrumentation when measuring coagulation parameters. Specifically, those that are in intensive care settings are often those that pose the greatest challenge. The authors should this as the next hurdle they will need to overcome to show that AT levels can be determined in the presence of yet more interference.

Response: Thank you very much for your insightful comments. The method utilizes an antibody to AT, which is generally considered highly specific and will not bind to other medications circulating in the blood. While additional medications may affect the extent of blood coagulation and, consequently, alter capillary flow velocity, we believe that such perturbations are negligible compared to the extent of particle-enhanced immunagglutination, given the 0.01% and 0.001% dilutions used in this study. A new paragraph is added to the Discussion section:

Thirdly, the assays were not tested in the presence of other medications, as the patients in intensive care settings often receive other drugs that may affect the extent of blood coagulation. The impact of these additional medications should be negligible, as the antibody to AT is generally considered highly specific and less likely to bind them. In addition, the capillary flow velocity change should primarily result from surface tension changes induced by particle immunoagglutination, as demonstrated in our previous work on the SARS-CoV-2 assay in human saliva [17]. Nonetheless, it will be worthwhile to include such results in the future, especially from patients’ samples in intensive care settings, to investigate the effects of various medications.

2. The statement in the introduction "Individuals who are predisposed to thrombotic events are likely to be ATIII-deficient" is misleading and inaccurate as there are many pathological reasons for a patient to be pro thrombotic and not just deficient in AT. Please revise this statement to reflect the general literature.

Response: The sentence was removed to avoid confusion. The new sentence directly discusses AT deficiency from the beginning:

… ~~Individuals who are predisposed to thrombotic events are likely to be ATIII-deficient, which could be hereditary or acquired over time.~~ AT deficiency could be hereditary or acquired over time. …

3. The section on ECMO seems out of place as AT do not be determined at the bedside, as this is done in a hospital laboratory. The rationale for rapid POC testing isn't presented in the paper for ECMO as daily monitoring of AT levels is common practice.

Response: All descriptions on ECMO were removed, focusing only on CPB.

4. Given the loading requirements for the human whole blood were diluted how will this be achieved at the bedside? Dilutions are prone to errors and often impossible to perform outside of a laboratory as the tools for performing them aren't at the bedside.

Response: Added a description of automated sample dilution in the Discussion section:

At this stage, diluting to 0.01% or 0.001% requires several steps of manual pipetting. While automated blood or plasma dilution is outside the scope of this proof-of-concept demonstration, numerous methods are currently available, including commercial sample dilution devices such as Dilumat® from bioMérieux [21] and microfluidic serial dilution devices [22].

New references are added:

21. bioMérieux. Dilumat®. 2024 [cited 22 September 2025].	Marcy-l’Étoile, France: bioMérieux. Available from: <https://www.biomerieux.com/us/en/our-offer/industry-products/dilumat-automated-sample-dilution.html>
22. Sugiura S, Hattori K, Kanamori T. Microfluidic serial dilution cell-based assay for analyzing drug dose response over a wide concentration range. Anal. Chem. 2010; 82: 8278-8282. <https://doi.org/10.1021/ac1017666>

5. Provide the details on how the dilutions were performed further to point 4.

Response: In the *Endogenous AT Levels in Human Blood Samples* sub-section of the Results:

… The undiluted sample from subject A was serially diluted at 10-fold increments to 0.001% and 0.01% concentrations, …

In the *AT and Blood Samples Tested* sub-section of the Materials and Methods:

… Serial dilutions were made at 10-fold increments to reach 0.01% or 0.001%.

6. In the statistical analysis, the R2 is good but the equation isn't discussed. Figure 4 shows a semi log transformation of the data to show a straight line which to this reviewer mean there is an exponential relationship between the time and the concentration of AT.

Response: Descriptions on the slope and y-intercept were added. The semi-logarithmic relationship has been demonstrated previously with the particle immunoagglutination assays using different detection methods.

In the *Capillary Flow Velocity Assay of AT in PBS* sub-section of the Results:

… with an R^2^ value of 0.988 (Figure 4B). The slope (0.136) indicates a semi-logarithmic relationship between capillary flow velocity and AT concentration, as previously demonstrated with the *E. coli* assays [12]. In fact, particle immunoagglutination assays have previously been shown to exhibit a similar semi-logarithmic relationship when monitored by light-scattering intensity [18] and by particle counting [16]. The y-intercept (0.575) represents the flow velocity in the absence of AT, i.e., the flow velocity with the particles not immunoagglutinated. …

A new reference was added:

18. Heinze BC, Gamboa JR, Kim K, Song JY, Yoon JY. Microfluidic immunosensor with integrated liquid core waveguides for sensitive Mie scattering detection of avian influenza antigens in a real biological matrix. Anal. Bioanal. Chem. 2010; 398: 2693-2700. <https://doi.org/10.1007/s00216-010-4201-y>

Additionally, previous Figures 5A and 5B – AT-spiked plasma assays – were merged into one (now Figure 6) to establish the standard curve. This standard curve was used to calculate AT concentrations from the newly collected human blood samples. This new standard curve was also discussed as shown below:

In Capillary Flow Velocity Assay of AT in Human Blood Plasma under Results:

Building on the initial calibration with PBS samples, we proceeded to test the assay’s ability to function in a complex matrix, diluted plasma. Figure 6A shows the results with diluted plasma containing varying amounts of spiked AT. At low AT concentrations, it shows the expected increasing trend similar to Figure 4A, corresponding to the left side of the bell-shaped curve (Figure 1B). At high AT concentrations, capillary flow velocities increased substantially, leading to a much steeper increase. For the given linear range, the curve was plotted against the logarithm of AT concentration, and a good linear relationship was observed, with a much higher slope (0.540 versus 0.136 in PBS) and an R² value of 0.973 (Figure 6B). Apparently, the presence of human blood plasma amplified the extent of particle immunoagglutination, potentially through minimizing non-specific immunoagglutination. However, this presumption could not be confirmed, as fluorescence staining and subsequent microscopic imaging of numerous plasma components are challenging on a paper substrate (because of their small sizes) [19].

Figure 6 (previously Figure 5) is modified to show the new “plasma” standard curve using AT-spiked plasma samples and the equation:

**Figure 6. Flow velocity assays with AT-spiked human blood plasma.** A) A human plasma sample with the endogenous AT level of 1.59 ng/mL was diluted to 0.001% and spiked with 0, 0.5, and 1 ng/mL AT, yielding 1.59, 2.09, and 2.59 ng/mL. It was also diluted to 0.01% and spiked with 0, 0.5, 1, 3, and 12 ng/mL AT, yielding 15.9, 16.4, 16.9, 18.9, and 27.9 ng/mL. Average flow velocities (pixel/frame) were plotted against AT concentration (n = 7 or 8). Error bars represent standard errors. B) The same plotted against logarithmic AT concentration to generate the standard curve equation.

The following is also added to the Discussion:

A logarithmic-linear relationship was observed in both AT-spiked PBS and diluted plasma samples, with very high R^2^ values of 0.988 and 0.973, respectively. The slopes of standard curves, on the other hand, were quite different: 0.136 in PBS and 0.540 in diluted plasma. The most abundant plasma protein is albumin, which has been widely used in immunoassays to passivate surfaces [8]. Such passivation minimizes non-specific antibody-antigen binding. In fact, the y-intercept of the standard curve is quite low, 0.040 with diluted plasma, compared to 0.575 with PBS. Such passivation of non-specific antibody-antigen binding maximizes the participation of antibody-conjugated particles in immunoagglutination, leading to high sensitivity and, consequently, a steeper slope in the standard curve. Albumin can also stabilize the antibody-conjugated particles, minimizing non-specific particle aggregation.

7. Could the authors comment about the use of such a device for other applications in coagulation where the clinical need is more urgent and the resources may not be available.

Response: A new paragraph was added to the Discussion to describe the other applications of this method.

This work can be adapted to quantify factors involved in blood coagulation, such as prothrombin time (PT), activated partial thromboplastin time (aPTT), thrombin time (TT), fibrinogen assay, von Willebrand factor (VWF) assay, anti-Xa assay, and lupus anticoagulant testing, among others [20].

A new reference was added:

20. Winter WE, Flax SD, Harris NS. Coagulation testing in the core laboratory. Lab. Med. 2017; 48: 295-313. <https://doi.org/10.1093/labmed/lmx050>

**Reviewer #2**

General Assessment: This manuscript presents a technically innovative proof-of-concept for detecting Antithrombin III (ATIII) using smartphone-based flow velocity measurements on paper microfluidic chips. While the concept is novel and potentially impactful, the current study does not sufficiently support its translation into a point-of-care (POC) diagnostic tool.

Major Comments
1. POC Applicability and Validation
The authors claim that this assay could fill a gap in POC testing for ATIII. However, this claim is not well substantiated:
• The assay is not validated against the immunoassays currently used in clinical diagnostics, such as Immunoassays/ELISA or functional activity assays.
• There is no formal method comparison (e.g., Bland–Altman analysis, regression, or bias estimation) between the smartphone-based assay and the ELISA results.
• The clinical relevance and interchangeability of the smartphone-based flow assay with existing diagnostic standards remain unclear.
Recommendation: Include a direct method comparison using patient samples across the clinical range, with appropriate statistical analysis of agreement and bias.

Response: While we included assay results from the commercial ELISA kit (Supplementary S5 File) and our smartphone-based assays (Figures 5 and 6), we acknowledge that the relationship between the ELISA and our smartphone assays is not clearly shown in the main text. To address the reviewer’s criticisms, we have renewed our IRB approval and recruited additional human subjects. We collected additional human blood samples from 12 new subjects, bringing the total to 14 (including previously included A and B). For each subject, dilutions were made separately, and three independent flow velocity assays and three independent ELISAs were conducted. Flow velocity assay data were augmented by a factor of 4 by collecting flow distances from 4 lines in each channel.

In Endogenous AT Levels in Human Blood Samples under Results:

… The total number of human subjects was 14. The first two subjects’ samples, labeled A and B, were used to construct a standard curve. The remaining 12 subjects’ samples, labeled 1 through 12, were used to correlate the flow-velocity assay with ELISA, as discussed later.

Figure 5 shows the ELISA assay results. Since the kit detects AT level in the ng/mL range, we diluted the samples to 0.01%. The standard curve is shown in A), using a series of AT solutions in PBS, ranging up to 50 ng/mL. With 0.01% plasma, the normal range corresponds to 15-30 ng/mL. The obtained concentrations were converted to 0.001% plasma concentrations to compare them with those from flow velocity assays adequately. The results are shown in B), showing that the majority of samples fall in the normal range of 1.5-3.0 ng/mL, or at least very close to it.

Figure 5 has been added to show the ELISA standard curve (previously in S5 file) and ELISA results for 14 human subjects (1-12 are new, and A-B were previously included in S5 file).

**Figure 5. ELISA assays of human plasma samples.** A) A standard curve using a series of AT solutions in PBS. Average of three measurements for each data point. B) AT concentrations in 0.001% plasma from 13 human subjects. Averages of three measurements for each subject. AT concentrations were calculated using the standard curve equation shown in A).

Previous Figures 5A and 5B – AT-spiked plasma assays – were merged into one (now Figure 6) to establish the standard curve. This standard curve was used to calculate AT concentrations from the newly collected human blood samples.

In Capillary Flow Velocity Assay of AT in Human Blood Plasma under Results:

Building on the initial calibration with PBS samples, we proceeded to test the assay’s ability to function in a complex matrix, diluted plasma. Figure 6A shows the results with diluted plasma containing varying amounts of spiked AT. At low AT concentrations, it shows the expected increasing trend similar to Figure 4A, corresponding to the left side of the bell-shaped curve (Figure 1B). At high AT concentrations, capillary flow velocities increased substantially, leading to a much steeper increase. For the given linear range, the curve was plotted against the logarithm of AT concentration, and a good linear relationship was observed, with a much higher slope (0.540 versus 0.136 in PBS) and an R² value of 0.973 (Figure 6B). Apparently, the presence of human blood plasma amplified the extent of particle immunoagglutination, potentially through minimizing non-specific immunoagglutination. However, this presumption could not be confirmed, as fluorescence staining and subsequent microscopic imaging of numerous plasma components are challenging on a paper substrate (because of their small sizes) [19].

Figure 6 (previously Figure 5) is modified to show the new “plasma” standard curve using AT-spiked plasma samples and the equation:

**Figure 6. Flow velocity assays with AT-spiked human blood plasma.** A) A human plasma sample with the endogenous AT level of 1.59 ng/mL was diluted to 0.001% and spiked with 0, 0.5, and 1 ng/mL AT, yielding 1.59, 2.09, and 2.59 ng/mL. It was also diluted to 0.01% and spiked with 0, 0.5, 1, 3, and 12 ng/mL AT, yielding 15.9, 16.4, 16.9, 18.9, and 27.9 ng/mL. Average flow velocities (pixel/frame) were plotted against AT concentration (n = 7 or 8). Error bars represent standard errors. B) The same plotted against logarithmic AT concentration to generate the standard curve equation.

A whole new section is added to the end of Results:

*Correlation Between Capillary Flow Velocity Assay and ELISA Using Multiple Human Plasma Samples*

To validate against the laboratory standard, we conducted capillary flow velocity assays on human plasma samples from multiple human volunteers. Human whole blood samples were centrifuged to obtain plasma, which was then diluted to 0.001% in PBS. Capillary flow velocity assays were conducted in an identical manner described above. Three independent assays were performed for each human subject’s sample. For each assay, flow distances were measured at four lines and averaged to ensure accurate readings. Therefore, the total dimension of data was 3 x 4 = 12 for each subject. The flow velocities from the first 35 time frames were collected. The standard curve equation obtained from spiked human plasma samples, y = 0.540x + 0.040 (Figure 6B), was used to calculate AT concentrations.

Figure 8A shows the correlation between the flow velocity and ELISA-measured AT concentrations in 0.001% plasma. The slope is 1.06 and is quite close to 1, indicating the strong correlation between the two assays. Several outliers are evident (circled), which can be attributed to 1) subject-to-subject variation in plasma composition affecting the capillary flow, 2) potential errors in ELISA (incomplete rinsing and/or incubation), 3) potential defects in paper chips (residual wax blocking capillary flow), etc. These factors will be revisited in the Discussion.

We repeated the flow-velocity assays using the higher antibody-conjugated particle concentration on the same set of human plasma samples. The flow velocities were noticeably higher at the higher particle concentration, so we introduced a universal correction factor of 1.5 to calculate AT concentrations correctly. Figure 8B shows the result. It shows improved correlation with fewer outliers, though the results are not substantially different from Figure 8A.

Added to the Discussion:

The flow velocity assay was compared with a commercial ELISA kit using multiple human subject samples. It showed linear correlation between the two assays, although several outliers were observed. Such outliers can be attributed to 1) variations in plasma samples, 2) errors in ELISA, and 3) defects in the flow velocity assay. Firstly, the composition of plasma samples should vary from subject to subject, including albumin and fibrinogen. Albumin should play an important role in passivating non-specific immunoagglutination, which should affect the flow velocity assay either positively or negatively. Fibrinogen is responsible for blood coagulation, which should directly impact the capillary flow velocity. While the dilution of 0.001% should decrease their concentrations substantially, e.g., 45 mg/mL albumin in undiluted plasma is equivalent to 450 ng/mL in 0.001% plasma, they are still sufficiently higher than the detection limits of capillary flow assays (pg/mL scale) [11,12] and can make substantial impacts on particle immunoagglutination and flow velocity assay. Secondly, ELISA is not perfect and prone to user error, such as inconsistent pipetting, incomplete rinsing, and suboptimal incubation. One human subject sample (#2) showed a very low AT concentration of 0.95 ng/mL, substantially lower than the normal range of 1.5-3.0 ng/mL in 0.001% plasma (= 15-30 mg/dL in undiluted plasma). However, the flow velocity assay reported much higher AT concentrations in both Figures 8A and 8B, creating the most obvious outlier at the far left, potentially indicating an error in the ELISA. Thirdly, the wax-printing process for fabricating paper chips may also be imperfect, as residual wax can remain in the main channel and subsequently block capillary action [16]. In such a case, the capillary flow velocity should be lower than expected, and the AT concentration should be underestimated. The outliers at the bottom-right in Figures 8A and 8B are potential examples.

New Figure 8 is added:

**Figure 8. Correlation between flow velocity- versus ELISA-measured AT concentrations.** A) AT concentrations were calculated using the standard curve shown in Figure 5B: y = 0.540x + 0.040. Averages from three measurements for each human subject sample. They are plotted against the ELISA concentrations shown in Figure 7. Samples tested: 1-12 and A. B) Higher concentrations of antibody-conjugated particles were used, and the flow velocities were measured (n = 3 per subject). The calculated AT concentrations were divided by a universal correction factor of 1.5. Samples tested: 1-12. For both A) and B): Averages from three different experiments for each sample, and four line distances per each experiment. Outliers are circled in both plots.

2. Claim of Filling a Diagnostic Gap
The manuscript suggests that this method fills a critical gap in ATIII testing. However, this is not convincing:
• Portable immunoassay platforms (e.g., lateral flow, microfluidic ELISA) are already being developed for multiple analytes and are more directly translatable to POC settings.
• The proposed method requires precise dilution, controlled environmental conditions, and smartphone-based video analysis, which may not be feasible in real-world point-of-care (POC) environments typically operated by non-laboratorians.
Recommendation: Temper the claim and discuss alternative POC strategies. Justify why this method offers a unique advantage over portable immunoassay devices.

Response: We acknowledge the current limitations of our method.

Firstly, as the other reviewer pointed out, our claim of potential use in ECMO was an overstatement. Therefore, we removed all ECMO-related descriptions.

Secondly, as the other reviewer also pointed out, we did not test our method in the presence of other medications, although we believe this should not substantially affect the results. A new paragraph is added to the Discussion section:

Thirdly, the assays were not tested in the presence of other medications, as the patients in intensive care settings often receive other drugs that may affect the extent of blood coagulation. The impact of these additional medications should be negligible, as the antibody to AT is generally considered highly specific and less likely to bind them. In addition, the capillary flow velocity change should primarily result from surface tension changes induced by particle immunoagglutination, as demonstrated in our previous work on the SARS-CoV-2 assay in human saliva [17]. Nonetheless, it will be worthwhile to include such results in the future, especially from patients’ samples in intensive care settings, to investigate the effects of various medications.

Thirdly, we acknowledge that dilution is a major bottleneck to this method’s use in POC settings. We added a description of automated sample dilution in the Discussion section:

At this stage, diluting to 0.01% or 0.001% requires several steps of manual pipetting. While automated blood or plasma dilution is outside the scope of this proof-of-concept demonstration, numerous methods are currently available, including commercial sample dilution devices such as Dilumat® from bioMérieux [21] and microfluidic serial dilution devices [22].

New references are added:

21. bioMérieux. Dilumat®. 2024 [cited 22 September 2025].	Marcy-l’Étoile, France: bioMérieux. Available from: <https://www.biomerieux.com/us/en/our-offer/industry-products/dilumat-automated-sample-dilution.html>
22. Sugiura S, Hattori K, Kanamori T. Microfluidic serial dilution cell-based assay for analyzing drug dose response over a wide concentration range. Anal. Chem. 2010; 82: 8278-8282. <https://doi.org/10.1021/ac1017666>

We removed most of the “Point of Care” from the title, abstract, keywords, and main text to avoid overstatement.

Title: Point of Care Antithrombin III … -> Rapid Antithrombin …

Keywords: Removed POC.

Abstract and Author Summary:

… Currently available methods for detecting AT levels are not portable, prompt, and simple enough to be used in urgent and clinical settings. …

Introduction:

… A portable, ~~POC~~ rapid, and simple test that can …

… ~~A POC~~ Such a test could immediately flag …

… A portable, rapid, simple ~~on-site~~ AT ~~POC~~ test would enable …

… ~~A rapid POC~~ Such a test to detect AT would be …

The overarching aim is to explore a rapid, easy-to-use, low-cost, and portable ~~POC~~ test that can be translated into real-world clinical and field applications.

Discussion:

This work aims to provide a novel proof-of-principle ~~POC~~ AT assay that utilizes changes in capillary flow velocity arising from particle immunoaggregation. …

Conclusion:

The work aims to fill a critical gap in the clinical need for a novel, ~~POC~~ portable, and rapid test for AT levels.

Further integration, automation, reproducibility, smartphone-to-smartphone variations, variations in environmental conditions, etc., are addressed in our response to comment 5, shown below.

3. LoD Determination and Method Evaluation
The manuscript mentions sensitivity and detection limits but does not describe how Limit of Detection (LoD) was formally determined.
• There is no mention of CLSI guidelines (e.g., EP17-A2, EP05-A3, EP09-A3) or any standard framework for evaluating analytical performance.
• LoD appears to be inferred from calibration curves, but without statistical justification or replicate data.
Recommendation:
• Clearly define how LoD was calculated (e.g., blank + 1.645 × SD of low concentration sample).
• State whether CLSI or other recognized guidelines were consulted.
• Discuss precision, reproducibility, and analytical range in the context of clinical diagnostics.
Additionally, the manuscript should address how this method compares to in vitro diagnostic standards in terms of:
• Analytical sensitivity and specificity
• Bias and agreement
• Sample preparation complexity
• Turnaround time and operational feasibility

Response: LOD can only be calculated using a negative sample. Since AT is present in all blood samples, including those from AT-deficient patients, there is no way to determine LOD. Calculating LOD on a low AT sample may not be the best way to evaluate assay performance.

We mentioned LODs for *E. coli* and Zika virus from our previous work, which included negative samples. To avoid confusion, modifications were made in the Introduction:

… Therefore, the capillary flow velocity should increase as the target antigen concentration increases. The limits of detection (LOD) were 10 CFU/mL for *E. coli* and 20 pg/mL for Zika virus. (LOD does not apply to AT assays, since AT is always present in blood samples, and negative samples do not exist.)

However, typical AT levels in plasma are 15-30 mg/dL (= 150-300 μg/mL) [13], several orders of magnitude higher than typical eNAMPT concentrations and 10^5^-10^7^-fold higher than ~~LODs~~ the typical assay ranges for *E. coli* and Zika virus.

The logarithmic-linear relationship was extended to AT-spiked plasma samples, and a new “plasma” standard curve was established in the modified Figure 6 (previously Figure 5). This curve shows the analytical range for plasma samples. Statistical analysis and parameter evaluation were newly added to the Discussion:

A logarithmic-linear relationship was observed in both AT-spiked PBS and diluted plasma samples, with very high R^2^ values of 0.988 and 0.973, respectively. The slopes of standard curves, on the other hand, were quite different: 0.136 in PBS and 0.540 in diluted plasma. The most abundant plasma protein is albumin, which has been widely used in immunoassays to passivate surfaces [8]. Such passivation minimizes non-specific antibody-antigen binding. In fact, the y-intercept of the standard curve is quite low, 0.040 with diluted plasma, compared to 0.575 with PBS. Such passivation of non-specific antibody-antigen binding maximizes the participation of antibody-conjugated particles in immunoagglutination, leading to high sensitivity and, consequently, a steeper slope in the standard curve. Albumin can also stabilize the antibody-conjugated particles, minimizing non-specific particle aggregation.

Due to the lack of negative samples, sensitivity and specificity cannot be defined. Nonetheless, we have newly added a comparison between the flow velocity assay and ELISA, as described in our response to comment 1. This new dataset can help determine how sensitive and specific our assay is.

Assay turnaround time, operational feasibility, sample preparation, and assay complexity are described in the Discussion:

Once the sample was loaded onto the paper chip, it took less than 2 minutes to reach the end of the channel. However, there is no need to wait until 2 minutes, since only the first 0-35 frames (1.17 seconds) of data were needed. As long as the antibody-conjugated particles are pre-loaded, dried, stored in a refrigerator, and provided to the experimenter, the assay can be conducted in near-real time (within seconds). The majority of assay time will be spent on plasma dilution. It only requires a paper chip (pre-loaded with antibody particles) and a smartphone, and involves only one pipetting step for sample loading. In other words, it requires neither expensive laboratory equipment nor extensive expertise to carry out the assay.

4. ELISA Kit Details (Line 219)
The manuscript references the use of a commercial ELISA kit to determine endogenous ATIII levels but lacks sufficient detail.
Recommendation: Provide:
• Full kit name, manufacturer, and catalog number (already partially listed as SimpleStep Human ATIII ELISA, Abcam).
• Lot number, calibration range, and sensitivity.
• Sample preparation and dilution protocols.
• Whether ELISA was used as a reference method for validation.

Response: We conducted ELISA on 12 additional human subjects and compared the results with those from the flow velocity assays. Please refer to our response to comment 1 and the newly added Figures 5 and 8.

Details about ELISA are added in Endogenous AT Levels in Human Blood Samples under Results:

… and their endogenous AT levels were determined using a commercial AT ELISA kit (SimpleStep Human Antithrombin ELISA kit, catalog number ab222507, Abcam Limited, Cambridge, UK). …

Modifications were made in AT and Blood Samples Tested under Materials and Methods:

Human whole blood and subsequent human plasma samples were acquired from human volunteers. The University of Arizona’s Institutional Review Board (IRB) approved the protocol, designated as STUDY00004578. AT was also spiked into the diluted human plasma and diluted human whole blood. Two different plasma dilutions were tested: 0.01% and 0.001%. One whole-blood dilution was used: 0.001%. Serial dilutions were made at 10-fold increments to reach 0.01% or 0.001%.

AT levels in human blood plasma and whole blood were quantified using a commercial AT ELISA kit, SimpleStep Human Antithrombin ELISA kit (catalog number ab222507; Abcam Limited, Cambridge, UK). This kit detects AT levels in the ng/mL range, with the lowest detectable concentration of 0.33 ng/mL. The standard curve and the calculation of endogenous AT levels can be found in Figure 5. Raw data of all ELISA tests can be found in the S5 file.

A new section is added to the end of Materials and Methods:

*Correlation Between Capillary Flow Velocity Assay and ELISA Using Multiple Human Plasma Samples*

Flow velocity assays were repeated with multiple human plasma samples (diluted to 0.001%) using the identical method described above. The standard curve equation of AT-spiked diluted human plasma samples (Figure 6) was used (y = 0.540x + 0.040) to calculate AT concentration. For each experiment, flow distances were measured at four lines and averaged to ensure accurate readings. Three plasma samples were tested for each subject, yielding a total of 12 data dimensions per subject. These flow-velocity assay results were plotted against ELISA concentrations, using two different antibody-conjugated particle concentrations (Figure 8). Raw flow profiles for all correlation assays are in the S6 and S7 files, and the corresponding processed flow velocities are in the S8 file.

5. Device Translation and Usability
The manuscript does not address how the smartphone-based flow assay would be translated into a deployable device for clinical use.
Recommendation:
• Describe the envisioned device architecture (e.g., integrated chip-reader, automated sample loading).
• Discuss challenges in standardizing flow velocity measurements across different smartphones and environmental conditions.
• Address reproducibility and robustness in field settings.

Response: The following paragraph is added to the end of the Discussion:

To apply this preliminary proof-of-principle for a clinical setting, several further studies and modifications may be necessary. While smartphone-to-smartphone variations should be minimal, since the method does not “quantify” optical density but rather monitors flow motion, the edge-detection parameter may need to be adjusted slightly depending on ambient lighting conditions. In a situation where smartphone use is forbidden, a smartphone can be replaced with a Raspberry Pi and a Pi camera, which can also be interfaced with cloud-based analysis. An automated sampling loading system can also make the system more user-friendly. An open-source automated pipette method can be used alongside the proposed method, including https://github.com/ac-rad/digital-pipette or the one demonstrated by our group [23].

A new reference is added:

23. You DJ, Yoon JY. Droplet centrifugation, droplet DNA extraction, and rapid droplet thermocycling for simpler and faster PCR assay using wire-guided manipulations. J. Biol. Eng. 2012; 6: 15. https://doi.org/10.1186/1754-1611-6-15

Minor Comments
• Figures: Consider adding a schematic comparing the smartphone-based assay to a conventional immunoassay workflow.
• Terminology: Clarify units and dilution factors consistently (e.g., ng/mL vs mg/dL).
• Data Presentation: Include calibration curves and raw flow velocity data in supplementary materials.

Response: While a schematic of conventional ELISA may be beneficial to the readers not familiar with basic analytical biochemistry, we believe citations to reference [8] should be sufficient.

As for units, we used ng/mL throughout the manuscript, which is standard in most analytical chemistry and biosensor research. However, most clinical work, especially plasma analysis, uses the mg/dL unit. We used mg/dL units only when referring to normal AT concentrations in undiluted plasma, and in such cases, we used ng/mL units on the diluted plasma side-by-side.

A new standard curve (calibration curve) for diluted plasma is shown in Figure 6 and used to calculate AT concentrations from the newly recruited human subjects (Figure 8).

All raw flow velocity data are included in S2, S6, and S7 files.

Conclusion
This manuscript presents an interesting and technically sound proof-of-concept. However, its current form does not support the claim of clinical POC applicability. Additional validation, comparative analysis, and discussion of device translation are needed to strengthen the manuscript and justify its relevance to clinical diagnostics.

Response: Thank you for your constructive criticisms. Please refer to our additions and modifications described above, along with the substantially modified Figure 6, and newly added Figure 5 and Figure 8.

Final Note: The title should be revised to remove the term “Point of Care,” as the current assay is not yet suitable for POC use. POC testing is typically performed by non-laboratorians, and ease of use, minimal sample preparation, and straightforward data interpretation are essential. These aspects are not adequately addressed in the current study.

Response: Please refer to our response to comment 2. Overstatement has been corrected, and current limitations have been addressed. POC was dropped or replaced throughout the manuscript, including the title, abstract, and keywords.
